# Popular Biofortified Cassava Cultivars Are Heavily Impacted by Plant Parasitic Nematodes, Especially *Meloidogyne* Spp.

**DOI:** 10.3390/plants9060802

**Published:** 2020-06-26

**Authors:** Aminat Akinsanya, Steve Afolami, Peter Kulakow, Elizabeth Parkes, Danny Coyne

**Affiliations:** 1 Department of Crop Protection, Federal University of Agriculture, P.M.B. 2240, Abeokuta 110001, Ogun State, Nigeria; steveafolami@gmail.com or; 2 International Institute of Tropical Agriculture, P.M.B. 5320, Oyo Road, Ibadan 200001, Oyo State, Nigeria; p.kulakow@cgiar.org (P.K.); e.parkes@cgiar.org (E.P.); d.coyne@cgiar.org (D.C.); 3 Nematology Research Unit, Department of Biology, Ghent University, 9000 Gent, Belgium

**Keywords:** carotenoid content, *Manihot esculenta*, nutrition, root-knot nematodes, storability

## Abstract

The development of new biofortified cassava cultivars, with higher micronutrient contents, offers great potential to enhance food and nutrition security prospects. Among the various constraints affecting cassava production are plant parasitic nematodes (PPN), especially root-knot nematodes. In this study, six popular biofortified cultivars were field-evaluated for their response to PPN in Nigeria. A field naturally infested with a diversity of PPN but dominated by root-knot nematodes was used. Application of the nematicide carbofuran significantly reduced PPN densities, and at harvest, no root galling damage was observed, compared with untreated plots, which had heavy galling damage. Plant height, stem girth, plant weight, marketable storage root number and weight were significantly lower for most cultivars in untreated plots. Percentage yield losses in the range of 21.3–63.7% were recorded from two separate trials conducted for 12 months each. Lower total carotenoid and dry matter contents were associated with higher PPN densities in some biofortified cultivars, resulting in a loss of as much as 63% of total carotenoid and 52% of dry matter contents. The number and weight of rotted storage roots were significantly greater in untreated plots across cultivars, reducing in-field and post-harvest storability. This study demonstrates that natural field populations of PPN can substantially affect yield, quality and nutritional value of released biofortified cassava cultivars.

## 1. Introduction

Cassava is a major staple food crop in tropical and subtropical Africa, Asia, and Latin America, where approximately 500 million people depend on it as a major carbohydrate (energy) source [1]. It is an important crop for food security in these regions, partly because it yields more energy per hectare than many other major crop. In Africa, cassava is the most important of all root and tuber crops as a source of calories for human and livestock needs, ranking 4th, after rice, sugarcane and maize [2]. To meet rising demands for cassava but also to improve its nutritional value as a food, there has been considerable investment to breed improved cultivars, including for higher mineral and vitamin contents, a process referred to as biofortification [3]. The specific enhancement of nutritional elements through genetic improvement is referred to as biofortification [4]. The Bill and Melinda Gates Foundation have supported a global effort to develop cassava germplasm enriched with bioavailable nutrients since 2005. The BioCassava Plus initiative has six major objectives, including reducing cyanogen content, delaying postharvest deterioration and developing disease-resistant cultivars [5]. Using hybridization and selective breeding, researchers in Nigeria have developed new yellow cultivars of cassava that naturally produce a higher level of beta-carotene, which will help in reducing malnutrition caused by vitamin A deficiency in the region [6]. A total of seven biofortified cassava cultivars with total carotenoid content in the range of 8–12 μg/g fresh weight and 30–33% dry matter have been released [5], which compare with white cultivar total carotenoid content and dry matter in the range of 0.05–0.09 μg/g fresh weight and 35–37%, respectively. The dry matter content for provitamin A cultivars, therefore, is relatively lower compared to locally used cultivars and is a priority for improvement [5]. Cassava biofortification has largely been aimed at addressing vitamin A deficiency [7], an important public health problem in sub-Saharan Africa.

However, while raising the nutritional value of storage roots is a worthy objective, with great prospects for impacting the lives of millions, it must be additionally coordinated and associated with other valuable traits, such as pest and disease resistance. A number of biotic constraints affect the production of cassava, especially diseases and in particular virus diseases [8]. Major efforts have focused on breeding resistance against these threats into new, improved cultivars [8]. Less recognized threats, however, such as PPN have received much less attention. Although not well recognized, there is considerable, and growing, evidence of the damage that PPN inflict on cassava production [9,10,11,12]. In many cases, however, nematode damage often goes unnoticed. Traditionally, cassava is considered a hardy crop and generally viewed as immune to PPN. The naturally ‘knobbly’ and rough texture of the roots, which can disguise nematode damage to casual observation, partly aids this perception, while there may also be few roots present at harvest, especially if affected by nematode infection and have become necrotic and died off. Among the PPN that infect cassava, root-knot nematodes (*Meloidogyne* spp.) are the most prominent [13,14]. A number of studies have demonstrated the damaging impact of root-knot nematode infection, including their association with an increased incidence of rots [14]. Other PPN are also associated with cassava losses, such as *Pratylenchus brachyurus* and *Scutellonema bradys*, but the root-knot nematodes *M. incognita* and *M. javanica* are the most commonly reported and the most important nematode pests [12]. Some reports have documented almost total losses due to *Meloidogyne* spp. [15], although the association is sometimes not always so clear [16]. The association with rots can also disguise nematode damage and losses attributable to PPN but is an important aspect. This indirect consequence can lead to greater losses due to secondary fungal and bacterial rots and indeed has been shown to be strongly associated [17]. Given that improving the storability of cassava storage roots is a key breeding objective, the appropriate management of PPN will undoubtedly contribute to improving this objective [15,17]. Earlier studies have illustrated the variable susceptibility of cassava cultivars to *Meloidogyne* spp. [14,16], including biofortified cultivars [14,18,19]. The current study was undertaken to evaluate the effect of PPN on officially released biofortified cultivars in the field. These cultivars, released in Kenya and Nigeria, had been reported in a previous study to be susceptible to *M. incognita* in pots [19]. Our study builds on the pot evaluation study; using a field naturally infested with PPN, we assessed the impact of PPN infection on these cultivars and the implication of this for cassava farmers and consumers in the region.

## 2. Results

### 2.1. Plant-Parasitic Nematode Identification and Densities

**Figure 1:**
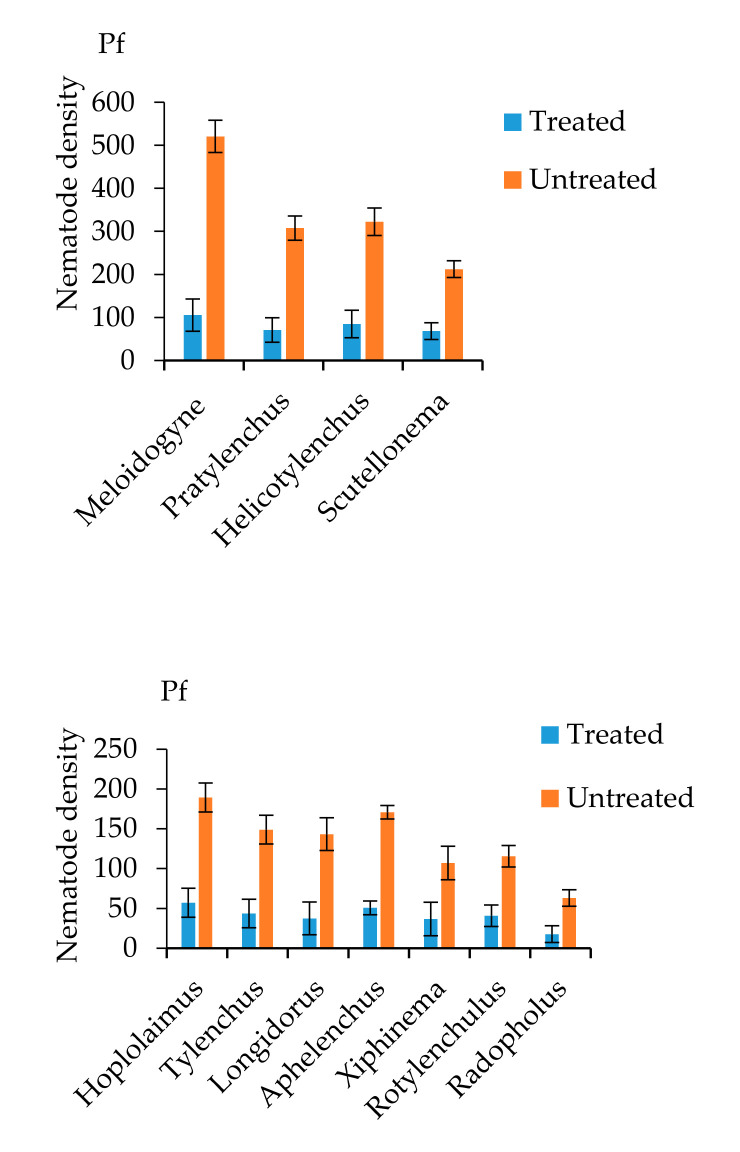
Effect of carbofuran on the final population of four major plant-parasitic nematodes encountered in field trials in Nigeria. P*f* = final density at harvest—12 months after planting; treatments: treated = 3G Carbofuran at 60 g/plot twice; untreated = untreated control. Error bars = Least Significant Difference (*p* ≤ 0.05).

Eleven genera of PPN were recorded: Meloidogyne, Pratylenchus, Helicotylenchus, Scutellonema, Hoplolaimus, Tylenchus, Longidorus, Aphelenchus, Xiphinema, Rotylenchulus and Radopholus (Figure 1). The initial population densities (Pi) were relatively low for seven nematode genera, while the more prominent genera were Meloidogyne, Pratylenchus, Helicotylenchus and Scutellonema (Table 1). Carbofuran application significantly (*p* ≤ 0.05) suppressed PPN densities in all treated plots, compared with the untreated plots (Figure 1). The genera Meloidogyne, Pratylenchus, Helicotylenchus and Scutellonema remained prominent over the duration of the trials with P*f*’s significantly (*p* ≤ 0.05) higher than other genera; Meloidogyne spp. had higher soil densities than all other genera (Figure 1).

### 2.2. Root Galling Damage and Host Suitability of Biofortified Cassava Cultivars Due to Meloidogyne Spp., 12 Months after Planting

All cultivars in the untreated plots reacted to *Meloidogyne* species infection with varying intensity, ranging from a gall index of 3.6–5.0 (Table 2). No galling of feeder roots was recorded in carbofuran-treated plots, but a low *Meloidogyne* population was recorded in the soil. A gall index of 5.0 was recorded on the check cultivar only, IITA-TMS-IBA30572, which also recorded the highest number of galls. Of the biofortified cultivars, IITA-TMS-IBA011368, IITA-TMS-IBA011371 and IITA-TMS-IBA070593 recorded the highest number of galls. In the untreated plots, all biofortified cassava cultivars reacted to *Meloidogyne* spp. infection and were all rated as good hosts, based upon a reproduction factor (RF) greater than 5.0 (Table 2).

### 2.3. Growth and Development of Biofortified Cassava Cultivars

The analysis of the data showed that there was no cultivar or treatment interaction per year at 6, 9 and 12 months after planting (MAP) (Table 3a). Therefore, the data from the experiments were pooled together for analysis (Table 3b). 

The application of carbofuran improved the growth of all cassava cultivars at some point during the growing cycle of the experiments; treated plants were generally significantly (*p* ≤ 0.05) taller and sturdier (Table 3b). Generally, stunting of aerial growth was observed on untreated plants at 3 MAP, which became more pronounced at 6, 9 and 12 MAP, when compared with treated plots. Cultivars were significantly (*p* ≤ 0.05) shorter in untreated plots compared with treated plots, except for IITA-TMS-IBA070593. Significant (*p* ≤ 0.05) reduction was also recorded in the stem girth of untreated plots in some of the cultivars, when compared with the treated plots. The overall mean showed that the growth and development of all cultivars in the untreated plots were significantly (*p* ≤ 0.05) suppressed at 3, 6, 9 and 12 MAP, when compared with treated plots, and the standard error (SE) was mostly higher in the untreated plots when compared with treated plots and increased at 3, 6, 9 and 12 MAP (Table 3b).

### 2.4. Yield Evaluation of Biofortified Cassava Cultivars

The results showed that there was cultivar and treatment interaction per year (Table 4a). The non-marketable storage yields showed no interaction per year (Table 4b), and these data were pooled together for analysis (Table 4e).

**Figure 2:**
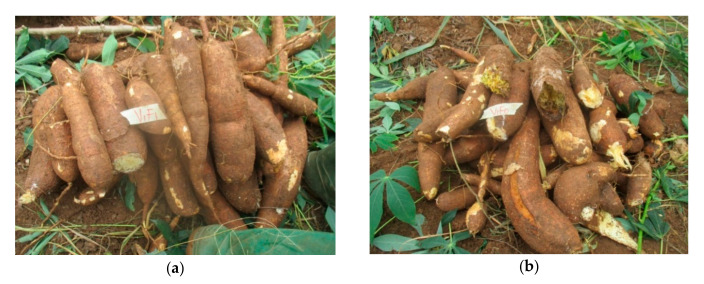
Storage roots of treated and untreated biofortified cassava cultivar IITA-TMS- IBA011368 at 12 months after planting; (**a**) treated with 3G Carbofuran; (**b**) untreated storage roots.

Plant weight and storage root yields were largely improved (*p* ≤ 0.001) across cassava cultivars in the two trials (Table 4a,c,d). Nematicide treatment led to higher (*p* ≤ 0.05) numbers and fresh weights of marketable storage roots in most cultivars, compared with untreated plots (Figure 2). The number and weight of non-marketable storage roots were significantly (*p* ≤ 0.05) lowered in cultivars IITA-TMS-IBA011368 and IITA-TMS-IBA011412 when compared with untreated plots in the first trial (Table 4c), while in the second trial (Table 4d), these cultivars in addition to NR 07/0220 were significantly (*p* ≤ 0.05) lowered. A significant (*p* ≤ 0.05) reduction in the number of rotted storage roots was also observed in treated plots, compared with untreated, with lower numbers for cultivars IITA-TMS-IBA011368 and NR 07/0220 (Table 4e). Fresh weights of rotted storage roots were similarly lower in treated plots, with cultivars IITA-TMS-IBA011368, NR 07/0220 and IITA-TMS-IBS30572 having significantly (*p* ≤ 0.05) less. The number and weight of deformed storage roots of cultivar IITA-TMS-IBA011412 were also less (*p* ≤ 0.05) in the treated plots, compared with the untreated (Table 4e). The overall mean showed that the aerial fresh weight of plants and the number and weight of marketable storage roots in the untreated plots were significantly (*p* ≤ 0.05) lower, when compared with treated plots in the two trials, while SE rates were higher in the untreated plots when compared with treated plots (Table 4c,d), while the number and weight of non-marketable (rotted and deformed) storage roots were significantly (*p* ≤ 0.05) lower in treated plots, with higher SE in the untreated plots when compared with treated plots (Table 4e).

Results showed that two biofortified cassava cultivars, IITA-TMS-IBA070593 and IITA-TMS-IBA070539, had significantly (*p* ≤ 0.05) lower total carotenoid contents of roots from untreated plants when compared with treated plants (Table 5). Similarly, dry matter content from untreated plants was lower in cultivars IITA-TMS-IBA011368 and IITA-TMS-IBA070593. When assessing total carotenoid and dry matter contents at the plot scale, however, taking into consideration the contents and yields, all the biofortified cultivars had significantly (*p* ≤ 0.05) lower values, compared with treated plots. The overall mean showed that total carotenoids per plot and dry matter per plant and per plot were significantly (*p* ≤ 0.05) lower in untreated plots, while SE rates were higher in the untreated plots when compared with treated plots (Table 5).

## 3. Discussion

A pot study using the same six biofortified cultivars as in the current study found them all to be good hosts to the root-knot nematode *M. incognita*, which reduced growth and development after six months in the screenhouse [19]. Although a number of other PPN genera were encountered, the majority were in relatively low densities and likely posed little threat to the cassava. Four genera were more prominent, of which *Meloidogyne*, the most important nematode genera attacking cassava, dominated the PPN community. The focus of the current study therefore centered on *Meloidogyne* spp., although it is understood that *Pratylenchus, Helicotylenchus* and *Scutellonema* spp. could have had some influence on cassava growth, which can become important when they are present in large densities [23]. The effect of *M. incognita* on the nutritional content of these biofortified cassava was less conclusive, but the study provided an indication that *M. incognita* infection can negatively impact cassava quality. The current study clearly supports the pot study findings but now also demonstrates that *Meloidogyne* spp. infection will reduce the nutritional value of improved, biofortified cassava under field conditions. Although the effect varied across cultivar and quality was not consistently reduced proportionally (per unit weight), by taking the yield impact into account, the overall damaging effect of *Meloidogyne* spp. can be better appreciated. All the tested cassava cultivars were susceptible to *Meloidogyne* spp. infection, resulting in significant (*p* ≤ 0.05) root galling damage and a reduction in plant growth and storage root yield of all but one of the six biofortified cultivars. Along with PPN densities, rotted storage roots were also much reduced in nematicide-treated plots. Rotting of storage roots directly affects their in-field storability, as well as their post-harvest longevity. Therefore, placing more emphasis on the management of PPN may be well justified, especially given the emphasis placed on nutritional biofortification and that increasing the storability and longevity of storage roots is a key breeding trait [16,24]. Carbofuran, however, is a toxic carbamate pesticide, which affects a wider range of pests and diseases than PPN alone. The reduction of rot-causing pathogens therefore is likely an additional effect, which would additionally reduce potential rots of cassava roots and should be considered. The pesticide did, however, enable a suitable comparison of PPN field densities, creating a differential against which to assess their impact on cassava. Other studies that have sought to assess the effect of PPN on cassava yield have used similar techniques, in addition to other methods, such as solarization [12,18,25]. From these studies some sizeable yield reductions have been associated with PPN, in particular *Meloidogyne* spp., demonstrating the need for their management if cassava production systems are to be sustainably intensified. Low yields have consistently characterized cassava production in Nigeria and other sub-Saharan countries. Nematode management may provide a major way forward in improving yields in farmers’ fields. The association between root rot incidence and *Meloidogyne* spp. infection has also been well demonstrated on cassava [18,25], as well as other root and tuber crops [16,26]. There is no doubting therefore the value of investing in PPN management and root-knot nematodes in particular, towards improving cassava productivity [12,14,15].

The current study showed that high PPN densities were associated with reduced crop performance following treatment with carbofuran, resulting in significant (*p* ≤ 0.001) yield loss of biofortified cassava. *Meloidogyne* spp. in the untreated plots caused galling on feeder roots of all biofortified cultivars. In Nigeria, *Meloidogyne* spp. infection caused significant (*p* ≤ 0.05) suppression in the growth and yield of elite cassava cultivars after 12 months in the field, despite relatively low observed levels of the nematode [14]. The loss in cassava yield was, however, mainly attributed to direct damage of the root system by the feeding activities of *Meloidogyne* spp. Although the current study was conducted at the International Institute of Tropical Agriculture (IITA) station, no inoculation was undertaken, natural PPN infestation levels were used and the trials were managed relative to farmer conditions. It is assumed, therefore, that these results provide a relatively fair reflection of the likely losses that farmers would experience. Elsewhere in Nigeria, significant cassava yield losses have also been recorded in farmer field trials naturally infested with *Meloidogyne* spp. Up to 200% yield increases were observed following the reduction of *Meloidogyne* spp. using solarization [25]. In Uganda, severe galling due to *Meloidogyne* spp. was reported in farmers’ fields [27]. Separately, 94% of fields examined in Uganda presented galling damage, with 17% severely affected, indicating substantial yield losses [28]. The impact of *Meloidogyne* spp. on cassava production is a threat to production that is likely to become increasingly acute and more intense under more intensified cropping conditions [11,14,15]. Besides reducing crop growth, vigor and productivity, PPN can reduce the quality and nutritional value of crop products. This is not surprising as PPN infect the root system, disrupting nutrient uptake and reducing their distribution within the plant [29,30,31]. PPN parasitize plants, changing the nutrient apportioning and cause disturbance in water and nutrient relations necessary for optimal plant growth [32]. Although a number of studies on various crops have indicated or demonstrated this, the empirical evidence is relatively limited. In our study, the total carotenoid and dry matter contents per plot of all biofortified cultivars were significantly (*p* ≤ 0.05) lower in untreated plots with higher PPN densities than treated plots. The current study and the preliminary pot study [15] now clearly show the impact on nutrition that *Meloidogyne* spp. can have, both on an individual plant, but especially when multiplied at scale. For example, the carotenoid content of cultivar IITA-TMS-IBA070539 was less by 0.19 kg per plot in untreated plots. This equates to a loss of 63% of total carotenoid content in the yield and quality of biofortified cassava, seriously undermining the efforts and investment to develop these high content biofortified cultivars.

Our study further confirms earlier reports that *Meloidogyne* spp. are the most prevalent and abundant PPN affecting cassava in Southwestern Nigeria. In the current study, the *Meloidogyne* spp. were not identified to species level, although it is likely that *M. javanica* and/or *M. incognita* were present, both of which are common to the region [33] and are the two most commonly occurring *Meloidogyne* spp. found infecting cassava [23]. As resistance against *Meloidogyne* spp. can be bred for in cassava, it would appear a useful mechanism for improving cassava for more intensive cultivation. The presence and infection of cassava by *Meloidogyne* spp. will reduce the yield and quality of cassava, including the nutritional content of biofortified cassava. Furthermore, *Meloidogyne* spp. infection is additionally associated with higher levels and incidence of rots, reducing the storability of cassava. PPN infection and damage to cassava has largely been overshadowed by other pests and diseases but is, however, a considerable threat to both yield, quality and storability. Breeding or actively selecting for nematode resistance during the evaluation process may therefore be more warranted than generally acknowledged or appreciated. In addition to creating more durable cultivars, more suitable to intensified cropping conditions, indirectly, this is likely to improve in-ground storability.

## 4. Materials and Methods

### 4.1. Experimental Details and Layout

Two field trials were planted in June 2017 and May 2018 in a well-drained sandy loam soil after ploughing and harrowing once each, at the IITA Ibadan, Nigeria (120 km north of Lagos). Cassava stems ~15 cm long were planted at an angle into the ground, spaced 1 × 1 m in a line 7 m long for each cultivar, representing a plot of 8 plants. Trials were maintained for 12 months after planting (MAP) before harvesting. The study consisted of two factors, cassava genotype (seven cultivars) and nematicide treatment (two levels). Six biofortified cassava cultivars (IITA-TMS-IBA011368, IITA-TMS-IBA011412, IITA-TMS-IBA011371, IITA-TMS-IBA070593, IITA-TMS-IBA070539 and NR 07/0220) and a check cultivar of white cassava (IITA-TMS- IBA30572) were obtained from the IITA. The nematicide 3G Carbofuran was applied at the rate of 3 kg a.i./ha (60 g/plot) at planting and repeated at 3 MAP and compared with a control receiving no nematicide. The experiment was laid out in a randomized complete block design with four replicates each per cultivar per treatment.

### 4.2. Assessment of Nematode Population Density and Damage

Soil samples were collected from 8 points per plot using a soil auger to a depth of 30 cm at planting to obtain initial nematode population density (P*i*) and at harvest to obtain final nematode population density (P*f*). Nematodes were extracted from 250 g soil sub-samples using the tray method [34], after removing all stones and debris and thoroughly mixing the bulked soil from each plot. At harvest, roots from 5 plants per plot were combined, gently tapped free of soil, chopped finely, thoroughly mixed and a 10 g sub-sample removed for nematode extraction using the same method as for soil. Nematode extracts were removed after 24 h, allowed to settle for 5 h and the volume adjusted to 30 mL by siphoning off the excess [35]. The mean nematode density was assessed under a compound microscope from 5 x 1 mL aliquots pipetted into a Doncaster counting dish [36]. Nematodes were identified to genus level using Bell’s Key [37] and a multiple tally counter used to count the different nematode genera. Total number of nematodes per plot from soil and root data was used to calculate the nematode reproduction factor (RF) [21]:

(1) $$\frac{Pf\;\times \;250\;g/\mathrm{soil}\;+\;Pf\;\times \;10\;g/\mathrm{root}}{Pi}$$

At harvest, the number of galls on 5 cm feeder roots per plant, removed randomly from 5 plants per plot, was counted and galling index (GI) per plant root assessed using the 1–5 gall index scale [20] (1 = 1–2 galls, 2 = 3–10 galls, 3 = 11–30 galls, 4 = 31–100 galls, 5 = > 100 galls).

#### Host Status

Host suitability was categorized as good when P*f*/P*i* > 5.0, fair if 5.0 ≥ P*f*/P*i* > 1, poor if 1 ≥ P*f*/P*i* > 0 and nonhost when P*f*/P*i* = 0 based on a study method [22].

### 4.3. Measurement of Crop Growth Parameters

Crop growth parameters were collected at 3, 6, 9 and 12 MAP for plant height and girth from five randomly selected cassava plants per plot. At harvest, the five selected plants per plot were additionally assessed as a bulk (plot) for aerial plant weight, number and weight of marketable and non-marketable storage roots. Plant height was measured to the tallest point of pre-harvested plants using a wooden ruler; girth was measured at 10 cm above the soil surface using a Vernier caliper. Stem and leaf material per plant was weighed together per plot and recorded as plant fresh weight. Harvested storage roots were sorted into non-marketable (small) and marketable storage roots. Deformed storage roots (physically twisted) and those affected by root rot were counted and weighed separately. Total yield was computed from all harvested marketable and non-marketable storage roots per plot.

### 4.4. Carotenoid and Dry Matter Analysis

The nutritional content of storage roots was assessed using total carotenoid nutrient and dry matter content following the procedure outlined in [15]. Cassava storage roots from each plot were randomly divided, one for fresh and the other for dried analysis for total carotenoid and dry matter content, respectively. The roots were chopped into ~0.5 cm^3^ cubes and 100 g sub-samples for each plot were randomly removed to determine the total carotenoid content using the iCheck™ method (BioAnalyt GmbH, Teltow, Germany). Total carotenoid content and dry matter were conducted for the first trial only due to the high cost of this procedure. For dry matter analysis, the 100 g fresh storage root cubes were oven-dried at 70 °C for 72 h, then milled to obtain a homogeneous powder, stored in moisture-free plastic containers and dry matter calculated for each cultivar [38]:

(2) $$\mathrm{Dry}\;\mathrm{matter}\;(\%)\;=\;\frac{\mathrm{Final}\;\mathrm{weight}}{\mathrm{Fresh}\;\mathrm{weight}}\;\times \;100$$

### 4.5. Statistical Analysis of Data

Data were subjected to a factorial analysis of variance (ANOVA) using SAS 9.4 [39] statistical package and means separated using least significant difference (LSD) at 5% level of probability. The data from the two experiments were pooled for analysis for those that recorded no cultivar or treatment interaction per year.

## 5. Conclusions

It is abundantly clear from the results that nematodes are a major constraint to cassava production. Root-knot nematode *Meloidogyne* spp. and the lesion nematode *Pratylenchus* spp. were the most common and important nematodes encountered from the study while *Helicotylenchus* spp., *Scutellonema* spp. and *Hoplolaimus* spp. could also become important when present in large numbers. All the biofortified cassava cultivars were susceptible and reacted to *Meloidogyne* spp. with varying intensity of root galling, ranging between 3.50 and 5.00 index. This was associated with a significant (*p* ≤ 0.05) reduction in above-ground fresh weight, plant height, stem girth, marketable storage root weight and number in most cultivars. The nutrient analysis clearly demonstrates the negative impact of PPN on the nutrient quality of biofortified cassava. Therefore, breeding and/or selecting for resistance against PPN, especially *Meloidogyne* spp., is here highlighted as highly necessary to achieve good yields and maintain nutrient quality in biofortified cassava. This has particular relevance under more intensified cropping conditions, which exaggerate soil and root borne constraints. Furthermore, the effect of root-knot nematode infection on the reduction of total carotenoid and dry matter contents should be investigated. Carbofuran was used to effectively manage PPN densities in the field in the current study, but it is an environmentally hazardous product that has been removed from the market in many places, even if it is systemic and not toxic to plants [40,41]. Synthetic pesticides are also often out of reach for resource-poor African farmers due to their high cost. Consequently, there is the need to work out effective and sustainable nematode control strategies in order to improve growth, yield and quality of biofortified cassava. Root-knot nematodes are highly pervasive pests, which are becoming increasingly problematic across tropical cropping systems and as such require particular attention from breeders.

## Figures and Tables

**Table 1:** Population of four major plant-parasitic nematodes encountered at planting in field trials in Nigeria ^1^.

Treatment ^2^	*Meloidogyne* ^3^	*Pratylenchus* ^3^	*Helicotylenchus* ^3^	*Scutellonema* ^3^
	Total Initial Population (P*i*)			
Carbofuran	59.86 ^a^	23.71 ^a^	36.79 ^a^	19.43 ^a^
Untreated	59.07 ^a^	25.07 ^a^	36.71 ^a^	18.43 ^a^

^1^ n = 8: means of four replications x two experiments; ^2^ 3G Carbofuran was applied at a rate of 60 g/plot twice; ^3^ soil densities from 250 g/soil; for each treatment group values within a column followed by a different letter are significantly (*p* ≤ 0.05) different.

**Table 2:** Root galling damage and host suitability of six biofortified cassava cultivars due to *Meloidogyne* spp, 12 months after planting in field trials in Nigeria ^1^.

Cultivar	Treatment ^2^	Total *Meloidogyne* Density (root + soil) ^3^	Gall Index ^4^	RF (P*f*/P*i*) ^5^	Host Status ^6^
IITA-TMS-IBA011368	Carbofuran	411 ^a^	0.0 ^a^		
	Untreated	2087 ^b^	4.3 ^b^	36.6	G
IITA-TMS-IBA011412	Carbofuran	455 ^a^	0.0 ^a^		
	Untreated	1688 ^b^	3.8 ^b^	27	G
IITA-TMS-IBA011371	Carbofuran	485 ^a^	0.0 ^a^		
	Untreated	2194	4.3 ^b^	43.4	G
IITA-TMS-IBA070593	Carbofuran	525 ^a^	0.0 ^a^		
	Untreated	2064 ^b^	4.3 ^b^	35.7	G
IITA-TMS-IBA070539	Carbofuran	599 ^a^	0.0 ^a^		
	Untreated	1781 ^b^	3.8 ^b^	29.7	G
NR 07/0220	Carbofuran	483 ^a^	0.0 ^a^		
	Untreated	1800 ^b^	3.6 ^b^	28.2	G
IITA-TMS-IBA30572 (check)	Carbofuran	251 ^a^	0.0 ^a^		
	Untreated	2361 ^b^	5.0 ^b^	42.6	G

^1^ n = 8: means of four replications x two experiments; ^2^ 3G Carbofuran was applied at a rate of 60 g/plot twice; ^3^ root and soil densities combined from 10 g/roots and 250 g/soil; ^4^ treated plots recorded no galling; gall index: 1 = 1–2 galls, 2 = 3–10 galls, 3 = 11–30 galls, 4 = 31–100 galls, 5 = > 100 galls [20]; ^5^ RF = nematode reproduction factor [21]; ^6^ host status was categorized as good (G) when P*f*/P*i* > 5.0, fair (F) if 5.0 ≥ P*f*/P*i* > 1, poor (P) if 1 ≥ P*f*/P*i* > 0, and nonhost (N) when P*f*/P*i* = 0 [22]; for each treatment group values within a column followed by a different letter are significantly (*p* ≤ 0.05) different.

**Table 3:** (**a**). Mean squares for growth and development of six biofortified cassava and one white cultivar in field trials in Nigeria. (**b**) Growth and development of six biofortified cassava cultivars in field trials in Nigeria ^1^.

(**a**)									
**Source**	**Df**	**Plant Height (cm**)				**Stem Girth (cm**)			
		3 MAP	6 MAP	9 MAP	12 MAP	3 MAP	6 MAP	9 MAP	12 MAP
Year	1	124.95	410.81	84.88	393.38	0.0015	0.00009	1.10 *	2.26 *
Replicate	3	121.96	513.91	1095.76	502.9	0.1600 *	0.44104 *	0.09	0.05
Cultivar	6	3564.50 ***	5790.16 ***	4530.87 ***	9944.81 ***	0.7353 ***	0.94247 ***	0.32	0.47
Treatment	1	12,539.84 ***	38,498.68 ***	82,889.28 ***	127,419.80 ***	0.7873 **	3.97509 ***	5.99 ***	6.56 ***
Cultivar * treatment	6	263.1	762.68	367.09	2058.23	0.0451	0.24967 *	0.17	0.04
Cultivar * year	6	105.58	892.17	383.49	1343.34	0.1689 *	0.23217	0.19	0.62 *
Treatment * year	1	13.23	805.18	324.02	2047.73	0.5144 *	0.07509	0.21	0.02
Error	87	151.04	467.32	819.3	1200.16	0.0576	0.11483	0.17	0.22
(**b**)									
**Cultivar**	**Treatment ^2^**	**Plant Height (cm**)				**Stem Girth (cm**)			
		3 MAP	6 MAP	9 MAP	12 MAP	3 MAP	6 MAP	9 MAP	12 MAP
IITA-TMS- IBA011368	Carbofuran	89.58 ^a^	180.21 ^a^	198.30 ^a^	279.45 ^a^	1.28 ^a^	2.12 ^a^	2.46 ^a^	2.86 ^a^
	Untreated	61.30 ^b^	125.21 ^b^	154.16 ^b^	188.10 ^b^	1.14 ^a^	1.86 ^a^	1.93 ^b^	2.26 ^b^
IITA-TMS-IBA011412	Carbofuran	97.29 ^a^	154.33 ^a^	196.06 ^a^	261.43 ^a^	1.53 ^a^	2.34 ^a^	2.68 ^a^	2.94 ^a^
	Untreated	65.13 ^b^	99.46 ^b^	132.21 ^b^	197.45 ^b^	1.29 ^a^	1.51 ^b^	1.83 ^b^	2.40 ^b^
IITA-TMS-IBA011371	Carbofuran	83.69 ^a^	169.83 ^a^	221.08 ^a^	266.39 ^a^	1.31 ^a^	2.25 ^a^	2.55 ^a^	2.91 ^a^
	Untreated	61.95 ^b^	138.66 ^b^	154.70 ^b^	203.90 ^b^	1.19 ^a^	1.94 ^a^	2.28 ^b^	2.59 ^b^
IITA-TMS-IBA070593	Carbofuran	62.95 ^a^	146.20 ^a^	185.55 ^a^	214.50 ^a^	1.04 ^a^	1.94 ^a^	2.51 ^a^	2.96 ^a^
	Untreated	52.96 ^a^	125.44 ^a^	144.78 ^b^	190.73 ^a^	1.03 ^a^	1.60 ^a^	2.24 ^a^	2.56 ^b^
IITA-TMS-IBA070539	Carbofuran	66.33 ^a^	144.23 ^a^	181.95 ^a^	235.83 ^a^	1.46 ^a^	1.71 ^a^	2.53 ^a^	3.03 ^a^
	Untreated	50.60 ^b^	109.41 ^b^	129.30 ^b^	167.90 ^b^	1.18 ^a^	1.48 ^a^	2.04 ^b^	2.51 ^b^
NR 07/0220	Carbofuran	44.19 ^a^	109.68 ^a^	162.99 ^a^	201.60 ^a^	0.83 ^a^	1.43 ^a^	2.14 ^a^	2.50 ^a^
	Untreated	29.81 ^b^	86.49 ^a^	110.10 ^b^	129.88 ^b^	0.70a	1.35 ^a^	1.84 ^b^	2.08 ^b^
IITA-TMS-IBA30572	Carbofuran	83.24 ^a^	146.34 ^a^	191.30 ^a^	266.95 ^a^	1.45 ^a^	2.11 ^a^	2.44 ^a^	2.94 ^a^
(check)									
	Untreated	57.36 ^b^	106.56 ^b^	126.11 ^b^	175.98 ^b^	1.33 ^a^	1.53 ^b^	1.93 ^a^	2.35 ^a^
Overall mean	Carbofuran	75.32 ^a^	150.12 ^a^	191.03 ^a^	246.59 ^a^	1.27 ^a^	1.99 ^a^	2.47 ^a^	2.88 ^a^
	Untreated	54.16 ^b^	113.03 ^b^	135.91 ^b^	179.13 ^b^	1.12 ^a^	1.61 ^b^	2.01 ^b^	2.39 ^b^
SE	Carbofuran	3.69	8.94	7.35	9.51	0.1	0.1	0.09	0.12
	Untreated	3.91	5.61	10.08	11.93	0.08	0.14	0.16	0.20

*, **, *** = mean squares significant at *p* ≤ 0.05, 0.01 and 0.0001 probability levels, respectively; MAP = months after planting. ^1^ n = 8: means of four replications x two experiments; ^2^ 3G Carbofuran was applied at a rate of 60 g/plot twice; MAP = months after planting; SE = standard error; for each treatment group values within a column followed by a different letter are significantly (*p* ≤ 0.05) different.

**Table 4:** (**a**) Mean squares for yield evaluation of six biofortified cassava and one white cultivar in field trials in Nigeria. (**b**) Mean squares for non-marketable yield evaluation of six biofortified cassava and one white cultivar in field trials in Nigeria. (**c**) Yield evaluation of six biofortified cassava cultivars in first field trial in Nigeria ^1^. (**d**) Yield evaluation of six biofortified cassava cultivars in second field trial in Nigeria ^1^. (**e**) Non-marketable yield evaluation of six biofortified cassava cultivars in field trials in Nigeria ^1^.

(**a**)							
**Source**	**Df**	**Plant Weight ^1^**	**Marketable Storage Roots ^1^**		**Non-Marketable Storage Roots ^1^**		**Total Yield/Plot**
		Fresh Weight (kg)	Number	Weight (kg)	Number	Weight (kg)	Storage Roots (kg)
Year	1	10.26 *	24.89 *	23.87 **	8.58 **	3.16 **	10.38 *
Replicate	3	0.26	4.64	2.32	2.53	0.24	4.4
Cultivar	6	13.41 ***	27.71 ***	14.93 ***	4.07 **	0.71 *	16.50 ***
Treatment	1	142.88 ***	183.09 ***	223.74 ***	40.08 ***	9.37 ***	162.48 ***
Cultivar * treatment	6	2.39	3.07	3.53 *	3.73	0.82 *	2.44
Cultivar * year	6	0.54	4.99	0.7	3.64**	0.24	1.16
Treatment * year	1	0.84	10.81	0.01	0.44	1.16 *	1.35
Error	87	2.01	3.96	1.55	1.12	0.25	1.98
(**b**)							
**Source**	**Df**	**Rotted Storage Roots ^1^**		**Deformed Storage Roots ^1^**			
		Number	Weight (kg)	Number		Weight (kg)	
Year	1	1.75	0.97 *	2.89 *		0.79 **	
Replicate	3	0.67	0.29	1.5		0.09	
Cultivar	6	1.36	0.36	0.82		0.04	
Treatment	1	22.32 ***	6.51 ***	2.29		0.17	
Cultivar * treatment	6	1.99 *	0.48 *	0.58		0.1	
Cultivar * year	6	0.33	0.12	2.18 *		0.11	
Treatment * year	1	0.14	0.79 *	0.32		0.12	
Error	87	0.74	0.18	0.61		0.07	
(**c**)							
**Cultivar**	**Treatment ^2^**	**Plant Weight ^3^**	**Marketable Storage Roots^3^**		**Non-Marketable Storage Roots^3^**		**Total Yield/Plot**
		Fresh Weight (kg)	Number	Weight (kg)	Number	Weight (kg)	Storage Roots (kg)
**First trial**							
IITA-TMS-IBA011368	Carbofuran	6.68 ^a^	9.00 ^a^	7.05 ^a^	1.25 ^a^	0.25 ^a^	7.30 ^a^
	Untreated	3.50 ^b^	4.50 ^b^	2.23 ^b^	4.50 ^b^	1.93 ^b^	3.70 ^b^
IITA-TMS-IBA011412	Carbofuran	4.58 ^a^	7.23 ^a^	5.65 ^a^	1.25 ^a^	0.23 ^a^	5.88 ^a^
	Untreated	2.88 ^a^	3.25 ^b^	2.43 ^b^	5.00 ^b^	1.85 ^b^	3.60 ^b^
IITA-TMS-IBA011371	Carbofuran	5.40 ^a^	5.00 ^a^	6.08 ^a^	1.00 ^a^	0.18 ^a^	6.25 ^a^
	Untreated	3.30 ^b^	3.25 ^a^	2.28 ^b^	1.50 ^a^	0.73 ^a^	3.00 ^b^
IITA-TMS-IBA070593	Carbofuran	3.95 ^a^	4.75 ^a^	3.50 ^a^	0.75 ^a^	0.30 ^a^	3.65 ^a^
	Untreated	2.00 ^a^	2.50 ^a^	2.80 ^a^	0.50 ^a^	0.15 ^a^	3.10 ^a^
IITA-TMS-IBA070539	Carbofuran	4.48 ^a^	8.25 ^a^	4.88 ^a^	0.75 ^a^	0.23 ^a^	5.10 ^a^
	Untreated	2.30 ^b^	5.25 ^a^	2.10 ^b^	1.75 ^a^	0.75 ^a^	2.85 ^a^
NR 07/0220	Carbofuran	2.73 ^a^	6.00 ^a^	3.05 ^a^	0.75 ^a^	0.30 ^a^	3.35 ^a^
	Untreated	1.78 ^a^	1.75 ^b^	0.70 ^a^	1.75 ^a^	1.00 ^a^	1.68 ^a^
IITA-TMS-IBA30572 (check)	Carbofuran	5.32 ^a^	8.75 ^a^	4.93 ^a^	1.25 ^a^	0.60 ^a^	5.53 ^a^
	Untreated	3.28 ^b^	6.25 ^a^	2.95 ^a^	1.25 ^a^	0.85 ^a^	3.80 ^b^
Overall mean	Carbofuran	4.73 ^a^	7.00 ^a^	5.02 ^a^	1.00 ^a^	0.30 ^a^	5.29 ^a^
	Untreated	2.72 ^b^	3.82 ^b^	2.21 ^b^	2.32 ^b^	1.04 ^a^	3.10 ^b^
SE	Carbofuran	0.4	0.58	0.28	0.28	0.05	0.36
	Untreated	0.43	0.78	0.7	0.48	0.22	0.48
(**d**)							
**Cultivar**	**Treatment ^2^**	**Plant Weight ^3^**	**Marketable Storage Roots ^3^**		**Non-marketable Storage Roots ^3^**		**Total Yield/Plot**
		Fresh weight (kg)	Number	Weight (kg)	Number	Weight (kg)	Storage Roots (kg)
**Second trial**							
IITA-TMS- IBA011368	Carbofuran	7.55 ^a^	9.00 ^a^	8.33 ^a^	0.00 ^a^	0.00 ^a^	8.33 ^a^
	Untreated	3.75 ^b^	6.75 ^b^	4.20 ^b^	2.00 ^b^	1.05 ^a^	5.25 ^b^
IITA-TMS-IBA011412	Carbofuran	5.60 ^a^	6.00 ^a^	6.43 ^a^	0.75 ^a^	0.20 ^a^	6.63 ^a^
	Untreated	3.40 ^b^	6.25 ^a^	3.65 ^b^	1.75 ^a^	0.68 ^b^	3.33 ^b^
IITA-TMS-IBA011371	Carbofuran	6.23 ^b^	5.50 ^a^	6.05 ^a^	1.00 ^a^	0.10 ^a^	6.28 ^a^
	Untreated	3.50 ^a^	3.70 ^b^	3.70 ^b^	1.25 ^a^	0.20 ^a^	3.93 ^b^
IITA-TMS-IBA070593	Carbofuran	5.03 ^a^	6.75 ^a^	5.75 ^a^	0.50 ^a^	0.10 ^a^	5.85 ^a^
	Untreated	3.10 ^a^	4.50 ^a^	2.95 ^b^	1.50 ^a^	0.38 ^a^	3.33 ^a^
IITA-TMS-IBA070539	Carbofuran	5.43 ^a^	8.25 ^a^	5.55 ^a^	1.00 ^a^	0.33 ^a^	5.88 ^a^
	Untreated	3.25 ^a^	8.00 ^a^	3.03 ^b^	1.75 ^a^	0.55 ^a^	3.58 ^b^
NR 07/0220	Carbofuran	3.13 ^a^	7.50 ^a^	3.28 ^a^	0.50 ^a^	0.15 ^a^	3.43 ^a^
	Untreated	2.03 ^a^	4.25 ^b^	2.18 ^a^	2.25 ^b^	0.53 ^a^	2.70 ^a^
IITA-TMS-IBA30572	Carbofuran	5.63 ^a^	8.25 ^a^	6.35 ^a^	0.25 ^a^	0.13 ^a^	6.48 ^a^
(check)	Untreated	2.53 ^b^	4.25 ^a^	2.10 ^b^	0.10 ^a^	0.25 ^a^	2.35 ^b^
Overall mean	Carbofuran	5.51 ^a^	7.32 ^a^	5.96 ^a^	0.51 ^a^	0.14 ^a^	6.13 ^a^
	Untreated	3.08 ^b^	5.39 ^b^	3.12 ^b^	1.51 ^a^	0.52 ^a^	3.50 ^b^
SE	Carbofuran	0.45	0.6	0.23	0.22	0.09	0.42
	Untreated	0.52	0.81	0.92	0.54	0.26	0.64
(**e**)							
**Cultivar**	**Treatment ^2^**	**Rotted Storage Roots ^3^**		**Deformed Storage Roots ^3^**			
		Number	Weight (kg)	Number		Weight (kg)	
IITA-TMS-IBA011368	Carbofuran	0.00 ^a^	0.00 ^a^	0.63 ^a^		0.13 ^a^	
	Untreated	2.13 ^b^	1.09 ^b^	0.88 ^a^		0.28 ^a^	
IITA-TMS-IBA011412	Carbofuran	0.38 ^a^	0.06 ^a^	0.63 ^a^		0.15 ^a^	
	Untreated	1.75 ^a^	0.79 ^a^	1.50 ^b^		0.41 ^b^	
IITA-TMS-IBA011371	Carbofuran	0.50 ^a^	0.06 ^a^	0.25 ^a^		0.11 ^a^	
	Untreated	1.00 ^a^	0.49 ^a^	0.50 ^a^		0.15 ^a^	
IITA-TMS-IBA070593	Carbofuran	0.38 ^a^	0.05 ^a^	0.15 ^a^		0.05 ^a^	
	Untreated	0.38 ^a^	0.03 ^a^	0.51 ^a^		0.24 ^a^	
IITA-TMS-IBA070539	Carbofuran	0.25 ^a^	0.11 ^a^	0.63 ^a^		0.16 ^a^	
	Untreated	0.88 ^a^	0.33 ^a^	0.75 ^a^		0.29 ^a^	
NR 07/0220	Carbofuran	0.00 ^a^	0.00 ^a^	0.63 ^a^		0.20 ^a^	
	Untreated	1.13 ^b^	0.51 ^b^	1.00 ^a^		0.21 ^a^	
IITA-TMS-IBA30572	Carbofuran	0.13 ^a^	0.03 ^a^	0.25 ^a^		0.11 ^a^	
(check)	Untreated	0.63 ^a^	0.44 ^b^	0.50 ^a^		0.34 ^a^	
Overall mean	Carbofuran	0.23 ^a^	0.04 ^a^	0.45 ^a^		0.13 ^a^	
	Untreated	1.13 ^b^	0.53 ^b^	0.81 ^b^		0.27 ^b^	
SE	Carbofuran	0.12	0.03	0.23		0.07	
	Untreated	0.37	0.2	0.35		0.12	

(**a**) *, **, *** = mean squares significant at *p* ≤ 0.05, 0.01 and 0.0001 probability levels, respectively; ^1^ fresh storage roots number and weight in 5 plants/plot. (**b**) *, **, *** = mean squares significant at *p* ≤ 0.05, 0.01 and 0.0001 probability levels, respectively; ^1^ fresh storage roots number and weight in 5 plants/plot. (**c**) ^1^ n = 4: means of four replications; ^2^ 3G Carbofuran was applied at a rate of 60 g/plot twice; ^3^ fresh storage roots number and weight in 5 plants/plot; SE = standard error; for each treatment group values within a column followed by a different letter are significantly (*p* ≤ 0.05) different. (**d**) ^1^ n = 4: means of four replications; ^2^ 3G Carbofuran was applied at a rate of 60 g/plot twice; ^3^ fresh storage roots number and weight in 5 plants/plot; SE = standard error; for each treatment group values within a column followed by a different letter are significantly (*p* ≤ 0.05) different. (**e**) ^1^ n = 8: means of four replications x two experiments; ^2^ 3G Carbofuran was applied at a rate of 60 g/plot twice; ^3^ fresh storage roots number and weight in 5 plants/plot; SE = standard error; for each treatment group values within a column followed by a different letter are significantly (*P* ≤ 0.05) different.

**Table 5:** Nutritional quality of six biofortified cassava cultivars in field trial in Nigeria ^1^.

Cultivar	Treatment ^2^	Storage Roots Yield (kg/plot ^3^)	Total Carotenoid (µg/g fr.wt./plant ^3^)	Total Carotenoid (kg/plot ^3^)	Dry Matter (%/plant ^3^)	Dry Matter (kg/plot ^3^)
IITA-TMS-IBA011368	Carbofuran	7.30 ^a^	6.38 ^a^	0.23 ^a^	35.75 ^a^	2.61 ^a^
	Untreated	3.70 ^b^	7.38 ^a^	0.14 ^b^	30.25 ^b^	1.12 ^b^
IITA-TMS-IBA011412	Carbofuran	5.88 ^a^	6.58 ^a^	0.19 ^a^	33.25 ^a^	1.96 ^a^
	Untreated	3.60 ^b^	6.45 ^a^	0.12 ^a^	30.50 ^a^	1.10 ^b^
IITA-TMS-IBA011371	Carbofuran	6.25 ^a^	7.80 ^a^	0.24 ^a^	29.50 ^a^	1.88 ^a^
	Untreated	3.00 ^b^	8.13 ^a^	0.12 ^b^	30.00 ^a^	0.89 ^b^
IITA-TMS-IBA070593	Carbofuran	3.65 ^a^	9.81 ^a^	0.18 ^a^	34.50 ^a^	1.26 ^a^
	Untreated	3.10 ^a^	6.37 ^b^	0.10 ^a^	19.75 ^b^	0.61 ^b^
IITA-TMS-IBA070539	Carbofuran	5.10 ^a^	11.71 ^a^	0.30 ^a^	35.75 ^a^	1.82 ^a^
	Untreated	2.85 ^a^	7.93 ^b^	0.11 ^b^	37.75 ^a^	1.08 ^b^
NR 07/0220	Carbofuran	3.35 ^a^	5.06 ^a^	0.08 ^a^	24.25 ^a^	0.81 ^a^
	Untreated	1.68 ^a^	7.03 ^a^	0.06 ^a^	24.50 ^a^	0.41 ^b^
IITA-TMS-IBA30572 (check)	Carbofuran	5.53 ^a^	0.04 ^a^	0.00 ^a^	38.75 ^a^	2.14 ^a^
	Untreated	3.80 ^b^	0.00 ^a^	0.00 ^a^	36.25 ^a^	1.38 ^b^
Overall mean	Carbofuran	5.29 ^a^	6.77 ^a^	0.18 ^a^	33.11 ^a^	1.78 ^a^
	Untreated	3.10 ^b^	6.18 ^a^	0.09 ^b^	29.85 ^b^	0.94 ^b^
SE	Carbofuran	0.36	0.77	0.05	1.49	0.12
	Untreated	0.48	0.76	0.08	2.17	0.19

^1^ n = 4: means of four replications; ^2^ 3G Carbofuran was applied at a rate of 60 g/plot twice; ^3^ fresh storage weight in 5 plants/plot; SE = standard error; for each treatment group values within a column followed by a different letter are significantly (*p* ≤ 0.05) different.
